# Pyridoxal-5′-phosphate-dependent enzyme GenB3 Catalyzes C-3′,4′-dideoxygenation in gentamicin biosynthesis

**DOI:** 10.1186/s12934-021-01558-7

**Published:** 2021-03-09

**Authors:** Shaotong Zhou, Xiaotang Chen, Xianpu Ni, Yu Liu, Hui Zhang, Min Dong, Huanzhang Xia

**Affiliations:** 1 School of Life Science and Biopharmaceutics, Shenyang Pharmaceutical University, Benxi, 117004 China; 2 Frontiers Science Center for Synthetic Biology, Key Laboratory of Systems Bioengineering (MOE), School of Chemical Engineering and Technology, Tianjin University, Tianjin, 300072 China

**Keywords:** Gentamicin, C-3′,4′-dideoxygenation, Phosphotransferase, Pyridoxal-5′-phosphate (PLP)-dependent enzyme

## Abstract

**Background:**

The C-3′,4′-dideoxygenation structure in gentamicin can prevent deactivation by aminoglycoside 3′-phosphotransferase (APH(3′)) in drug-resistant pathogens. However, the enzyme catalyzing the dideoxygenation step in the gentamicin biosynthesis pathway remains unknown.

**Results:**

Here, we report that GenP catalyzes 3′ phosphorylation of the gentamicin biosynthesis intermediates JI-20A, JI-20Ba, and JI-20B. We further demonstrate that the pyridoxal-5′-phosphate (PLP)-dependent enzyme GenB3 uses these phosphorylated substrates to form 3′,4′-dideoxy-4′,5′-ene-6′-oxo products. The following C-6′-transamination and the GenB4-catalyzed reduction of 4′,5′-olefin lead to the formation of gentamicin C. To the best of our knowledge, GenB3 is the first PLP-dependent enzyme catalyzing dideoxygenation in aminoglycoside biosynthesis.

**Conclusions:**

This discovery solves a long-standing puzzle in gentamicin biosynthesis and enriches our knowledge of the chemistry of PLP-dependent enzymes. Interestingly, these results demonstrate that to evade APH(3′) deactivation by pathogens, the gentamicin producers evolved a smart strategy, which utilized their own APH(3′) to activate hydroxyls as leaving groups for the 3′,4′-dideoxygenation in gentamicin biosynthesis.

**Supplementary Information:**

The online version contains supplementary material available at 10.1186/s12934-021-01558-7.

## Background

**Fig. 1:**
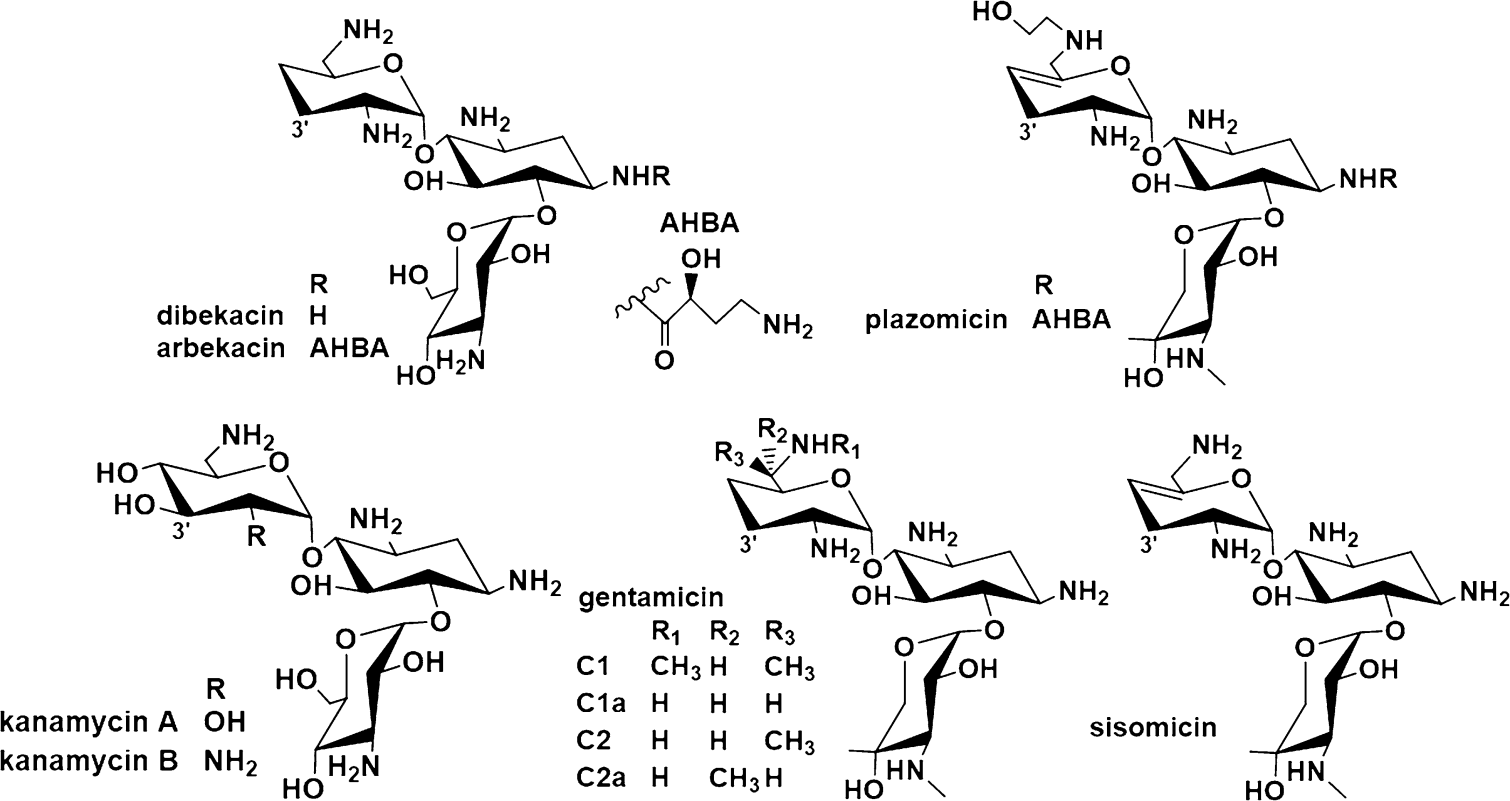
3′,4′-dideoxy and 3′,4′-hydroxyl aminoglycosides

Aminoglycoside antibiotics (AGAs) have been available for clinical use since the 1940s and have been shown to exhibit broad-spectrum activities [1]. However, the spread of resistance is limiting their use in the clinic, which is primarily induced by aminoglycoside-modifying enzymes (AMEs). The amino and hydroxyl groups of AGAs are deactivation targets of AMEs [2, 3]. Because deoxygenations remove the modifiable groups of AGAs, deoxy functionalities are natural defensive strategies that can prevent deactivation by AMEs [4]. The C-3′,4′-dideoxygenation structure can prevent deactivation by APH(3′) and adenyltransferase (ANT(4′)) [5]. Some semi-synthetic antibiotics have utilized the C-3′,4′-dideoxygenation structure to increase their efficacies, such as dibekacin (3′,4′-dideoxykanamycin B) [6], arbekacin (1-*N*-((*S*)-4-amino-2-hydroxybutyryl)-3′,4′-dideoxy-kanamycin B) [7, 8], and the newly approved sisomicin derivative plazomicin, which retains antibiotic activity in the presence of most AMEs [9, 10] (Fig. 1). The C-3′-deoxygenation biosynthetic process has recently been dissected by several research groups [11–13]. A radical S-adenosyl-L-methionine (SAM) enzyme, AprD4, along with AprD3, is responsible for the 3′-deoxygenation in apramycin biosynthesis [14]. The C-3′,4′-dideoxygenation biosynthetic process in gentamicin and sisomicin is still obscure.

**Fig. 2:**
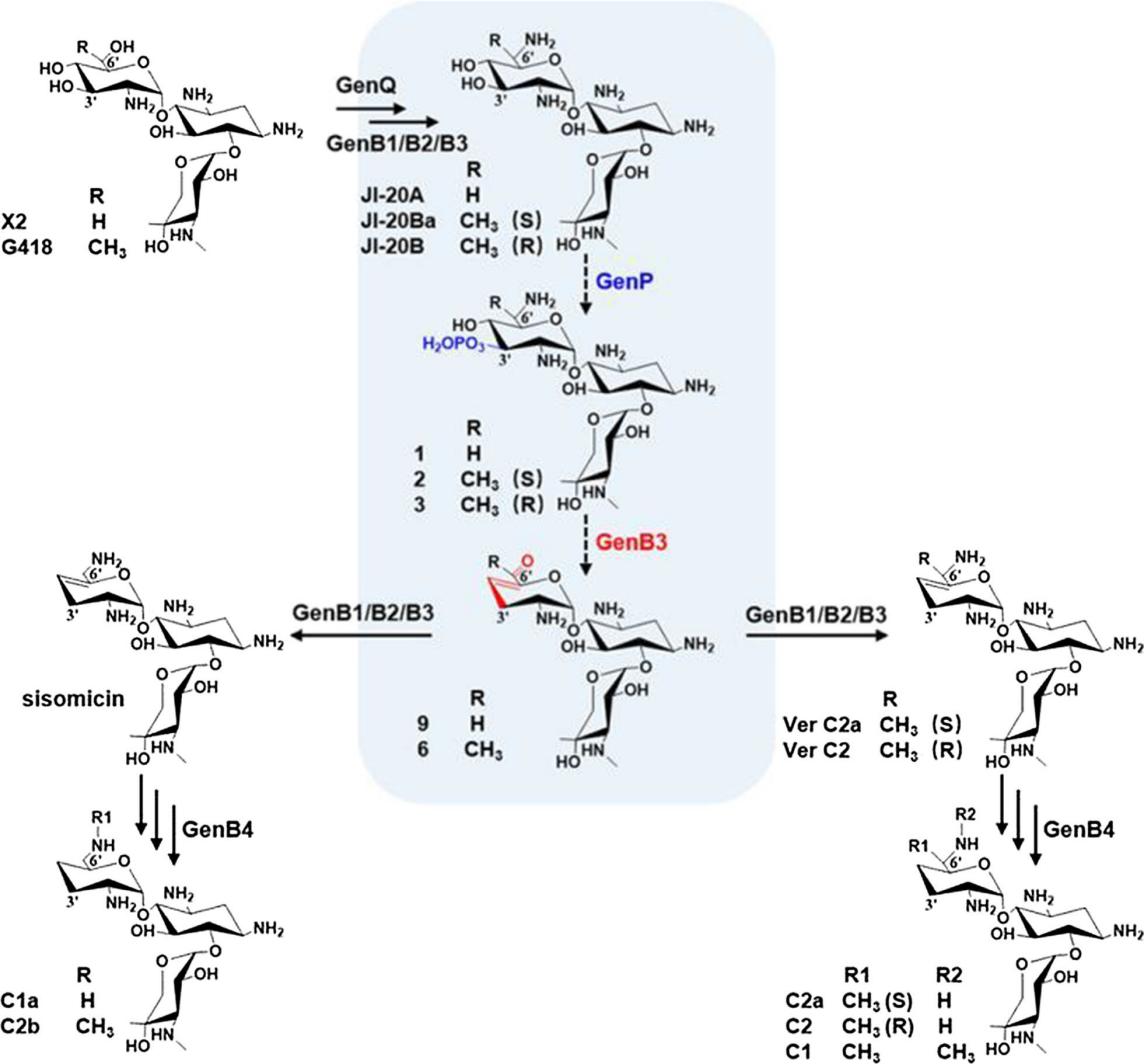
Biosynthetic pathway of gentamicin

Gentamicin is a broad-spectrum aminoglycoside bactericidal antibiotic produced by *Micromonospora echinospora* with a C-3′,4′-dideoxygenation moiety. As with other 2-deoxystreptamine-containing AGAs, significant progress has been made in the study of gentamicin biosynthesis [15–19]. The enzymatic steps that lead to the pseudotrisaccharide gentamicins JI-20A, JI-20Ba, and JI-20B have all been elucidated. They are the starting compounds of the C-3′,4′-dideoxygenation biosynthetic process. However, little detailed information of the featured C-3′,4′-dideoxygenation process is available (Fig. 2).

Genetic studies have shown that an APH(3′) GenP is involved in the aminoglycoside dideoxy process in gentamicin biosynthesis [20], and a biochemical study showed that GenP can phosphorylate C-3′ of an unnatural substrate, kanamycin B. Furthermore, kanamycin B is the synthetic precursor of dibekacin (3′,4′-dideoxy- kanamycin B) [21], so synthesis of dibekacin by C-3′,4′-dideoxygenation enzymes is a promising approach. GenP has 52% identity with ForP (GenBank No. CAF31545.1), while ForP can complement mutants in the dehydroxylation process of the fortimicin A biosynthesis pathway. Therefore, it is speculated that GenP can phosphorylate JI-20A, JI-20Ba, and JI-20B, which are the starting compounds of the C-3′,4′-dideoxygenation biosynthetic process [22].

Bioinformatic analysis showed that GenB3 is a PLP-dependent enzyme. Guo and colleagues showed that GenB3 catalyzes the 6′-transamination in the gentamicin biosynthetic pathway, although with lower activity than GenB1 and GenB2 [23] (Fig. 2). Interestingly, GenB3 has 85% identity with Sis18 (GenBank No. ACN38352.1) in the biosynthetic pathway of sisomicin, which is a 3′,4′-dideoxy-AGA too (Fig. 1). However, GenB3 has only approximately 30% identity with C-6′-aminotransferases in the biosynthetic pathway of AGAs without 3′,4′-dideoxygenation, like KanB [24] (GenBank No. CAF31586.1) from the kanamycin biosynthetic pathway (Additional file 1: Figure S1). Therefore, GenB3 may catalyze C-3′,4′-dideoxygenation in the gentamicin pathway.

In the present study, we demonstrate that the phosphorylation catalyzed by GenP is the first step in the gentamicin C-3′,4′-dideoxygenation process. More importantly, we show that a PLP-dependent enzyme, GenB3, uses the products of GenP to catalyze the dideoxygenation, forming 3′,4′-dideoxy-4′,5′-ene-6′-oxo products. The following transamination and reduction by GenB4 finish the gentamicin biosynthesis (Fig. 2).

## Results and discussion

### GenP starts the C-3′,4′-dideoxygenation process in the gentamicin biosynthetic pathway

**Fig. 3:**
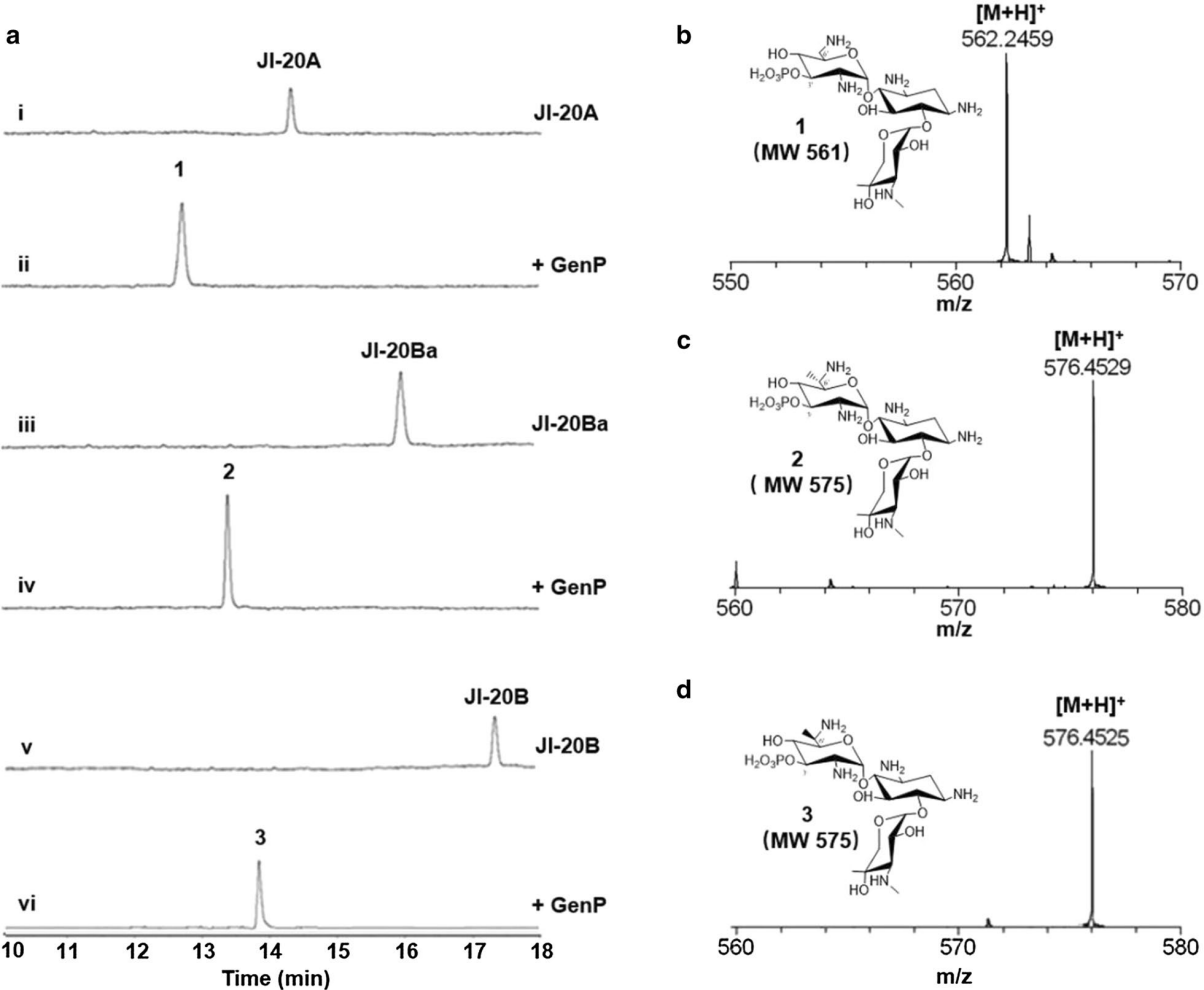
Catalytic activity of GenP. **a** HPLC-ELSD analysis of GenP reactions with gentamicin JI-20A, JI-20Ba, and JI-20B. **b**–**d** MS analysis of compounds **1**, **2**, and **3**. MW, molecular weight

To investigate the function of GenP in gentamicin biosynthesis, the intermediate metabolites of the biosynthetic pathway, JI-20A, JI-20Ba, and JI-20B were used as substrates. They are the starting compounds for the C-3′,4′-dideoxygenation process in gentamicin biosynthesis. The results of high-performance liquid chromatography using evaporative light-scattering detection (HPLC-ELSD) are shown in Fig. 3a. GenP converted all of these substrates into new products. Products of the reactions were analyzed by mass spectrometry (MS) and nuclear magnetic resonance (NMR) spectrometry. The ESI–MS spectrum suggested that GenP catalyzed the formation of monophosphorylated products (Fig. 3). Additionally, ^1^H, ^13^C, HHCOSY, and ^31^P NMR results of compound **2** confirmed that the product had a C-3′-OH phosphorylation moiety (Additional file 1: Figure S2). Further experiments showed that GenP had broad substrate specificities. In addition to phosphorylating the other two intermediate metabolites of the gentamicin biosynthetic pathway, X2 and G418, giving phosphorylated compounds **4** and **5** (Additional file 1: Figure S3), GenP can also phosphorylate kanamycin B, as previously reported [14]. However, GenP has a higher specificity toward JI-20A and JI-20Ba (Additional file 1: Table S1).

### GenB3 completes the C-3′,4′-dideoxygenation process

**Fig. 4:**
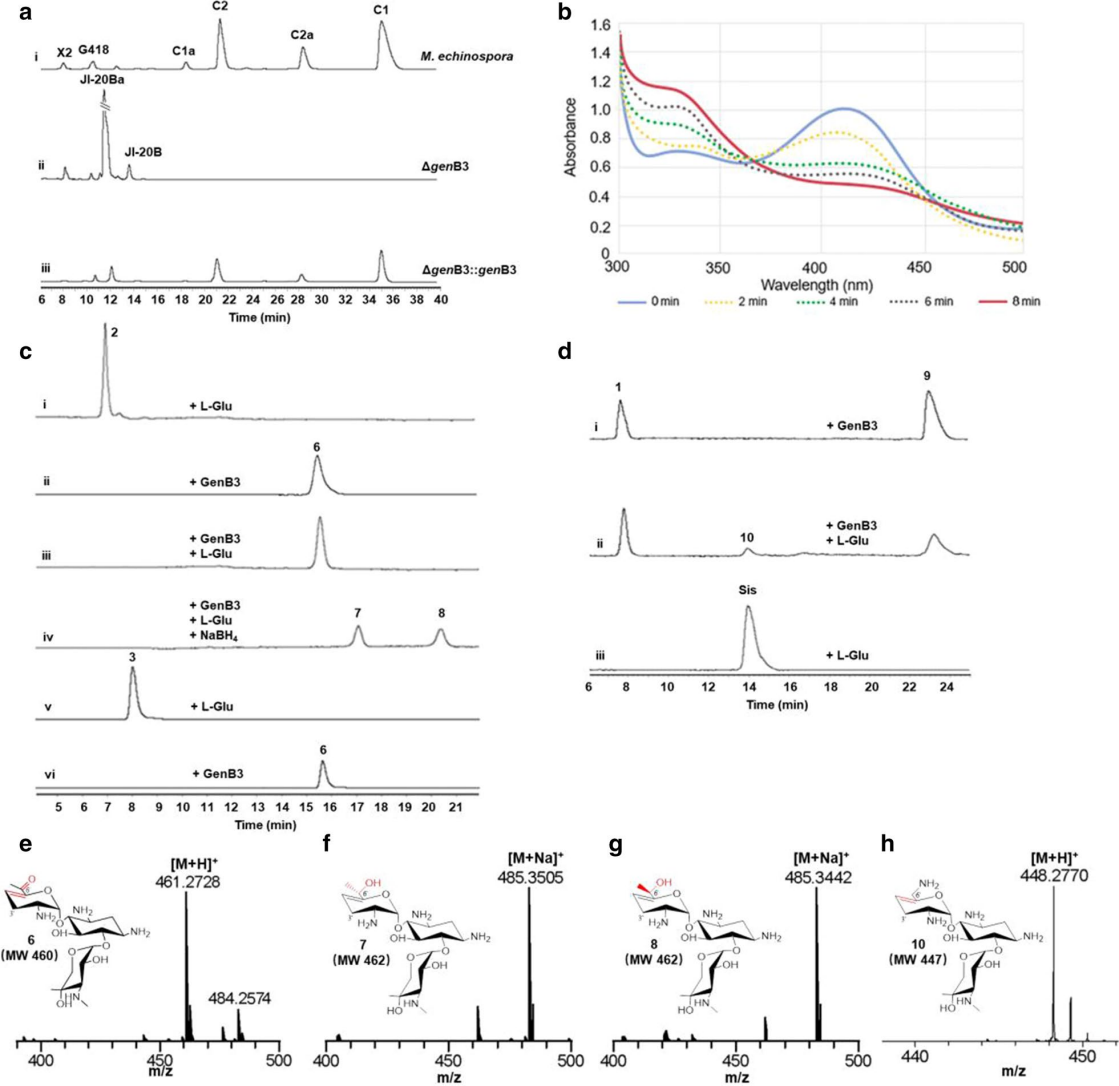
GenB3-catalyzed dideoxygenation. **a** HPLC-ELSD analysis of mutant- strain fermentation products, showing the (i) wild-type, (ii) *gen*B3-disruption strain (Δ*gen*B3), and (iii) Δ*gen*B3::*gen*B3 (complementation of the Δ*gen*B3 mutant). **b** Ultraviolet–visible absorption spectrum of GenB3-catalyzed reaction at different reaction times, where UV absorption was measured every 2 min from 300 to 500 nm. **c** HPLC-ELSD analysis of (i) the compound **2** standard with L-Glu, (ii) the GenB3-catalyzed reaction without L-Glu, (iii) the GenB3-catalyzed reaction with L-Glu, (iv) with NaBH_4_ added after the reaction, (v) the compound **3** standard with L-Glu, and (vi) the GenB3-catalyzed reaction with compound **3**. **d** HPLC-ELSD analysis. (i) The GenB3-catalyzed reaction with compound **1** was found to produce compound **9**. (ii) L-Glu was added. (iii) An L-Glu-added sisomicin standard (Sis) was used. **e**–**h** MS analysis of compounds **6**, **7**, **8**, and **10**

We then investigated the proteins with unknown function in gentamicin biosynthesis. To investigate the function of *gen*B3, gene-disruption experiments were performed. The disruption strain, Δ*gen*B3, did not produce C-3′,4′-deoxygenated products, but instead it accumulated non-deoxygenated products, including JI-20Ba and JI-20B (Fig. 4a). These results demonstrate that GenB3 is involved in gentamicin C-3′,4′-dideoxygenation.

To further investigate the role of GenB3, the recombinant protein was expressed and purified in *Escherichia coli*. GenB3 did not show obvious activity on JI-20Ba and JI-20B. We suspected that C-3′-phosphorylation-JI-20Ba (**2**) might be the substrate of GenB3 and the phosphate group could be hydrolyzed in the experiments with the Δ*gen*B3 strain. Indeed, HPLC-ELSD analysis showed that GenB3 converted **2** to a new compound **6** (Fig. 4c), which was characterized to be 6′-oxo-verdamicin by MS (Fig. 4e), ^1^H, and ^13^C NMR (Additional file 1: Figure S4). The results demonstrate that GenB3 catalyzed the C-3′-dephosphation and C-4′,5′-dehydratation of compound **2**. To gain more evidences on the keto group on C-6′, NaBH_4_ was added to the reaction mixture to reduce the keto group. HPLC-ELSD analysis showed that compound **6** disappeared after adding NaBH_4_, and two new peaks appeared as compounds **7** and **8** (Fig. 4c). MS analysis revealed that their molecular masses were both 462, and they were thus presumed to be C-6′-isomers of the hydroxyl group (Figs. 4f, g). In addition, JI-20Ba and JI-20B are a pair of C-6′ epimers, and GenB3 also catalyzes C-3′-phospho-JI-20B (**3**) to produce compound **6** (Fig. 4c).

When C-3′-phospho-JI-20A (**1**) was used as a substrate, GenB3 catalyzed C-3′-dephosphorylation and C-4′,5′-dehydration to form **9** (Fig. 4d). However, unlike with **6**, GenB3 further catalyzed the aminotransfer of C-6′ to form sisomicin (**10**) (Fig. 4h). This discrepancy may have been caused by the steric hindrance from the C-6′-methyl group of compound **6**. Results of UV spectrometry confirmed that PLP of GenB3 was converted to pyridoxamine 5′-phosphate (PMP) during the enzymatic reaction (Fig. 4b). Furthermore, no free ammonia was detected in the reaction solution (Additional file 1: Figure S5). In addition, addition of the amino acceptor α-ketoglutarate to the reaction mixture promoted the GenB3-catalyzed reaction (Additional file 1: Figure S6). These results indicate that the C-6′-amino group of the substrate was transferred to PMP.

All of the tested substrates of GenB3 contain amino groups at both C-2′ and C-6′. To identify which amino group is the functional group for PLP binding, we tested GenB3 activity toward substrates only containing one amino group at C-6′ and C-2′. GenB3 did not catalyze the deoxygenation of compounds containing the C-2′-amino group, but it did catalyze the deoxygenation of phosphorylated gentamicin B containing the C-6′-amino group. Therefore, the C-6′-amino group is the functional group for the GenB3-catalyzed reaction (Additional file 1: Figure S7).

### GenB3 first catalyzes the C-4′,5′-dehydration in the C-3′,4′-dideoxygenation process

**Fig. 5:**
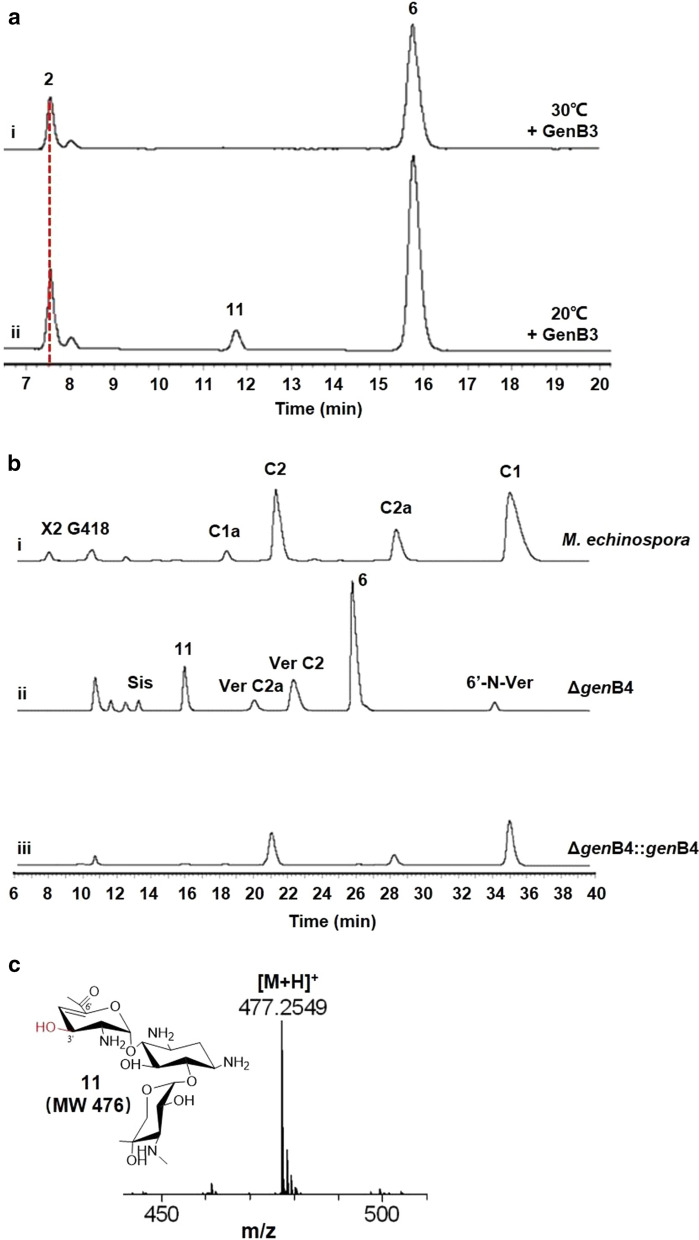
Discovery of **11** in GenB3-catalyzed reactions in vivo and in vitro. **a** HPLC-ELSD analysis of GenB3-catalyzed reactions in vitro with compound **2** at (i) 30 °C and (ii) 20 °C. **b** HPLC-ELSD analysis of *gen*B4-disruption strains identified compound **11**. **c** MS analysis of compound **11**

To further investigate the reaction mechanism of GenB3, the reaction condition was shifted from 30 to 20 °C, when C-3′-phospho-JI-20Ba (**2**) was used as a substrate. A small amount of compound **11** was detected in the reaction (Fig. 5a). MS results showed that it was a hydroxyl group at the 3′ position of **11** (Fig. 5c). We speculated that compound **11** was a reaction byproduct instead of an intermediate, because GenB3 did not further catalyze the 3′-hydroxyl deoxygenation of compound **11** with longer incubation times at 30 °C. We assumed that the C-4′,5′-dehydration occurred first in the dideoxygenation process, after which the C-3′-phosphate was removed. However, during unfavorable conditions for phosphate elimination, the C-3′-phosphate bond may have been hydrolyzed, which would then form compound **11**. This speculative step is consistent with our in vivo results. Compound **11** was also detected in *gen*B4-disruption strains (Fig. 5b). These results demonstrate that C-4′,5′-dehydration occurred first in the dideoxygenation process.

### Plausible mechanism of GenB3

**Scheme 1.**
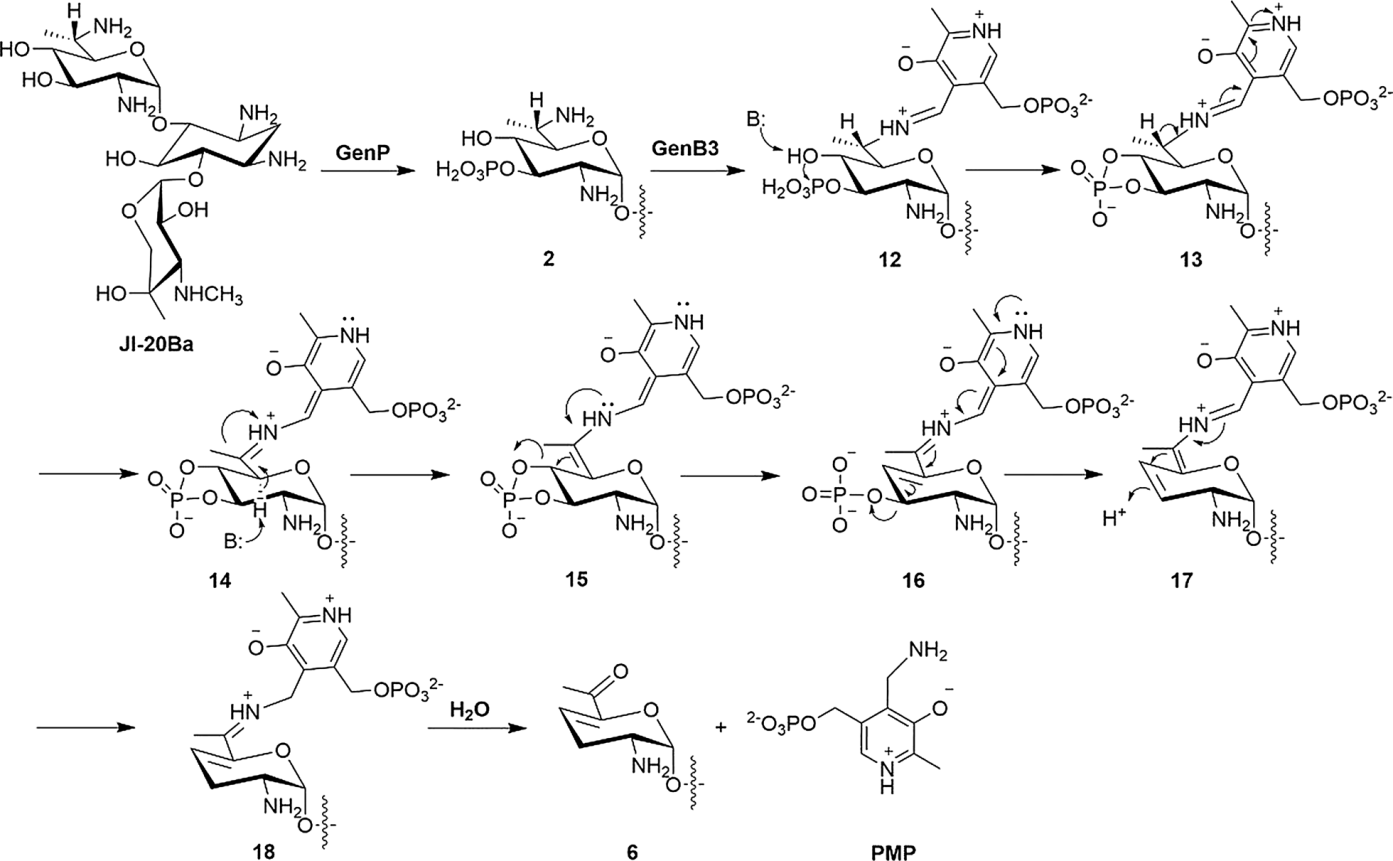
Proposed mechanism for the GenB3-catalyzed 3′,4′-dideoxygenation of JI-20Ba

Based on these results, we proposed the following mechanism for the GenB3 catalyzed dideoxygenation reaction (Scheme 1). Like other PLP-dependent enzymes, the reaction starts with an external aldimine **12** generated from the 6′-amine of compound **2** with the internal aldimine of the enzyme with PLP [25]. The 4′-hydroxyl group of aldimine **12** may be deprotonated by a basic residue and then attacks the adjacent phosphate group on the 3′-C, forming a more stable cyclic phosphodiester. By this means, the 4′-hydroxyl was activated by the enzyme as a good leaving group, which is a prerequisite for γ-elimination. A following deprotonation would convert the external aldimine **13** to the quinonoid **14** [26]. Then a classical PLP-dependent γ-elimination can happen, giving the 4′-deoxyl-4′,5′-olefin quinonoid **16** [27–29]. The 3′-phosphate group facilitates the second dehydroxylation as a good leaving group to yield the aldimine **17**. Protonation at 3′-C of the aldimine **17** affords ketimine **18**. Hydrolysis of the ketimine **18** would give the keto product compound **6** and PMP as the cofactor. This mechanism was supported by the isolation of the 3′-hydroxyl intermediate compound **11** from the GenB3 reaction, which should be the hydrolysis product of intermediate **16**.

GenB3-catalyzed products need further aminotransfer and reduction to produce components of gentamicin C. Although the intact C-6′-aminotransferases GenB1, and GenB2 existed in the *gen*B4-disruption strain, the main product of the *gen*B4-disruption strain was the 6′-oxo-containing compound **6** (Fig. 5b), which indicated that the C-6′-aminotransferase was inefficient for compound **6** in vivo*.* We have also proved that GenB4 could convert C-6′-amino-containing **6** (verdamicin C2 and verdamicin C2a) into gentamicin C2a, when GenB4 coupled with aminotransferases [23, 30]. However, when we incubated **6** with purified GenB4, the C-4′,5′-olefin reduction was undetectable via HPLC-ELSD (Additional file 1: Figure S8). These results demonstrate that the C-6′-amino group was a prerequisite for GenB4-catalyzed C-4′,5′-olefin reduction.

## Conclusions

In summary, the results presented here firmly demonstrate the functions of both GenP and GenB3 in gentamicin biosynthesis. GenB3 appears to be the first reported PLP-dependent enzyme catalyzing dideoxygenation in aminoglycoside biosynthesis. Interestingly, structurally related C-3′-deoxygenation and C-3′,4′-dideoxygenation of AGAs are catalyzed by distinct catalytic pathways. C-3′-deoxygenation is catalyzed through a radical mechanism by the radical SAM dehydratase, AprD4, along with the reductase partner, AprD3 [11–14]. Although an AprD3 homologue was identified in the gentamicin pathway, GenB3-catalyzed dehydration and 3′-phosphate elimination do not require a reductase partner. Instead, this process behaves in an “aminotransferase-like” manner, which is similar to that with dehydratase ColD from the biosynthetic pathway of l-colitose [31, 32]. PLP-dependent amino-transferases GenB1, GenB2, GenB3, and GenB4 not only have the common promiscuous activity to catalyze C-6′-aminotransfer as reported [23], but also have their unique functions in gentamicin biosynthesis, which demonstrates the diversity of PLP chemistry in enzymatic catalysis.

AMEs exist in both resistant pathogens and AGA producers [33]. It has been speculated that these enzymes may perform other metabolic functions [22]. In the present study, we have demonstrated that gentamicin producers evolved a smart strategy to evade APH(3′) deactivation by pathogens. The gentamicin biosynthetic pathway utilizes its APH(3′) to activate hydroxyls as leaving groups, and then the PLP-dependent enzyme GenB3 catalyzes dideoxygenation. The unveiling of the C-3′,4′-dideoxygenation pathway of gentamicin may pave the way for dissection of other sugar dideoxygenation pathways. As fortimicin and istamycin share the same C-3′,4′-dideoxygenation with gentamicin, and their biosynthetic gene clusters have identical enzymes with GenP and GenB3/GenB4 [34], their deoxygenation pathways may be identical with one another.

The APH(3′)-activated dideoxygenation pathway of gentamicin provides inspiration for chemical regiospecific removal of oxygen from sugars [35]. Since GenP, GenB3, and GenB4 catalyze C-3′,4′-dideoxygenation, they have the potential to be applied in combinatorial biosynthesis of C-3′,4′-dideoxygenation containing semi-synthetic antibiotics, such as dibekacin and arkekacin [36], to develop novel drugs against life-threatening pathogens and to yield an improved safety profile [37]. Because C-3′, C-4′, and C-6′ are frequently used chemical modification sites for semi-synthesis of AGAs [38–40], GenP, GenB3, and GenB4 may be used for combined chemical and enzymatic syntheses of promising therapeutic leads.

## Methods

### Strains and culture conditions

*Escherichia coli* Top10 was used as cloning host; *E. coli* ET12567 (pUZ8002) was used for *E. coli*–*M. echinospora* conjugation; *E. coli* BL21 (DE3) was used for protein expression. *E. coli* strains were grown in LB medium (tryptone 10 g/L, yeast extract 5 g/L, NaCl 10 g/L) at 37 °C via antibiotic selection (100 μg/ml ampicillin, 50 μg/ml apramycin, 25 μg/ml chloramphenicol, 50 μg/ml kanamycin). *Pfu* DNA polymerase was obtained from Vazyme, while GC buffer and dNTPs were obtained from Takara. T4 DNA ligase and DNA marker were purchased from Takara. *M. echinospora* ATCC 15,835 was used for the creation of in-frame deletion mutants and as the source of GenP (GenBank No. AGB13904.1) and GenB3 (GenBank No. AGB13905.1). The GenBank No. of the *M. echinospora* ATCC 15,835 genome is JQ975418.1. Gene sequencing was performed by Genewiz. *M. echinospora* ATCC 15,835 wild-type and mutants were grown in growth medium (soluble starch 10 g/L, wheat bran 10 g/L, MgSO_4_·7H_2_O 5 g/L, K_2_HPO_4_·3H_2_O 0.3 g/L, KNO_3_ 1 g/L, NaCl 0.5 g/L, CaCO_3_ 1 g/L, asparagine 0.02 g/L, agar 2.5 g/L) at 37 °C to obtain spores. For the collection of gentamicin, *M. echinospora* ATCC 15,835 wild-type and mutants were cultured in seed cultures (soluble starch 15 g/L, soybean powder 1 g/L, glucose 1 g/L, KNO_3_ 0.5 g/L, CaCO_3_ 23 g/L) at 34 °C for 36 h. Fermentation cultures (soluble starch 50 g/L, soybean powder 35 g/L, glucose 15 g/L, corn powder 15 g/L, peptone 15 g/L, (NH_4_)_2_SO_4_ 0.5 g/L, KNO_3_ 0.5 g/L, CoCl_2_·6H_2_O 0.01 g/L, CaCO_3_ 26 g/L) were conducted for 120 h.

### Construction of gene-disruption plasmids

For gene disruption, about 2000 bp upstream and downstream of the gene were amplified from the genomic sequence (see list of primers used in the Supplemental Information). PCR products (94 °C for 5 min; 30 cycles of 94 °C for 1 min, 60 °C for 45 s, and 72 °C for 2 min; 72 °C for 10 min) were cloned into the *E. coli*–*M. echinospora* vector pKC1139 or pD2925 (a plasmid derived from pIJ2925) to obtain the gene-disruption plasmids pKCP (*gen*P) and pDB3 (*gen*B3). All of the plasmids were verified by sequencing (plasmids are shown in Additional file 1: Table S3).

### Gene disruption of *gen*P and *gen*B3 genes

To obtain the mutant strains of Δ*gen*P and Δ*gen*B3, the gene-disruption plasmids pKCP and pDB3 were introduced into the wild-type strain by conjugation from *E. coli* ET12567 (pUZ8002, the gene disruption plasmid) to *M. echinospora.* Apramycin-resistant (Apr^R^) colonies were screened and confirmed by PCR amplification. The Apr^S^ colonies were selected from the initial Apr^R^ colonies and confirmed by PCR amplification (Additional file 1: Figure S9).

### Gene complementation of Δ*gen*P and Δ*gen*B3

Complementation plasmids contained the complete fragment of the gene under the control of the P_*hrd*B_ promoter. The complementation plasmids were prepared by cloning *gen*P and *gen*B3 into pEAP1 (a plasmid derived from pSET152) under the control of the P_*hrd*B_ promoter. The complementation plasmid was introduced individually into the mutant strain by conjugation. Complemented strains were based on erythromycin resistance (100 μg/ml) and were confirmed by PCR amplification.

### Extraction, isolation, and purification of gentamicin C complexes and intermediates

Fermentation products of wild-type and mutant strains were adjusted to a pH of 2.0 with H_2_SO_4_. After centrifugation (5,000 rpm, 10 min, room temperature), the supernatant was adjusted to a pH of 7.0 with NaOH. The fermentation broth was centrifuged again, and the supernatant was adsorbed by cationic resin D152 adsorption at 37 °C for 3 h. The adsorbed resin was packed into a column. The column was washed with 10 times the column volume of 0.01 mM to 0.2 M NH_3_·H_2_O. The eluate was freeze-dried, re-dissolved in 1 ml of water, and filtered through a 0.22 μm microporous membrane before subjection to HPLC-ELSD analysis.

### Construction of gene-expression plasmids in *E. coli*

*gen*P and *gen*B3 genes were amplified from the DNA of *M. echinospora* by PCR amplification (see the list of primers used in Additional file 1: Table S2). The PCR products were digested with *Nde*I and *Hin*dIII and were inserted into pET28a(+) to obtain gene-expression strains. Each plasmid was transformed into *E. coli* BL21 (DE3). The expression plasmids were verified by DNA sequencing.

### Expression and purification of GenP and GenB3

*E. coli* BL21 (DE3) containing the expression plasmid was cultured in LB (50 μg/ml kanamycin) at 37 °C to an OD_600_ of 0.6–0.8 and gene expression was induced by isopropylthiogalactoside (IPTG, 0.1 mM) at 16 °C for 20 h. The cells were collected by centrifugation and resuspended in 20 ml of binding buffer (0.5 M NaCl, 20 mM Tris–HCl at a pH of 8.0). The recombinant protein was obtained by sonication for 20 min (3 s on, 5 s off), and the supernatant was obtained by centrifugation at 4 °C for 20 min. The recombinant protein was purified by Ni^2+^-charged His-Bind resin (GE Healthcare). Impurities were eluted by washing buffer A (0.5 M NaCl, 20 mM Tris–HCl, 20 mM imidazole), washing buffer B (0.5 M NaCl, 20 mM Tris–HCl, 40 mM imidazole), and washing buffer C (0.5 M NaCl, 20 mM Tris–HCl, 60 mM imidazole). The recombinant proteins were eluted by elution buffer (0.5 M NaCl, 20 mM Tris–HCl, 200 mM imidazole). Imidazole in the eluted proteins was removed by dialysis. The purified proteins were stored at − 30 °C.

### Preparation of substrates for in vitro catalytic reactions

Different reaction substrates were obtained by fermentation of different mutant strains. Gentamicin X2 was obtained from the fermentation broth of the *M. echinospora* Δ*gen*KΔ*gen*Q mutant strain. G418 was purchased from Sigma. JI-20A was isolated from the fermentation broth of Δ*gen*KΔ*gen*P. JI-20Ba and JI-20B were isolated from the fermentation broth of *M. echinospora* Δ*gen*B1Δ*gen*P. Ver C2a was isolated from the fermentation broth of *M. echinospora* Δ*gen*B4. For the separation of these compounds, refer to Method 5.

### GenP-catalyzed reaction with JI-20A, JI-20Ba, and JI-20B

The GenP activity assays were conducted using mixtures containing substrate (200 μM), GenP (15.8 μM), ATP (10 mM), and MgCl_2_ (10 mM) in 500 μl Tris–HCl buffer (50 mM, pH 8.0). Incubations were at 37 °C for 1 h and were quenched by addition of chloroform (500 μl), followed by centrifugation to remove proteins. The supernatants were filtered through 0.22 μm microporous membranes before subjection to HPLC-ELSD analysis.

### Separation and purification of compounds **1** and **2**

In order to obtain large amounts of compounds **1** and **2**, the volume of the GenP-catalyzed reaction was expanded to 10 mL, including substrates (JI-20A or JI-20Ba, 4 mM), GenP (25 μM), ATP (100 mM), MgCl_2_ (50 mM), and Tris–HCl buffer (50 mM, pH 8.0). Samples were incubated at 30 °C overnight. The reaction was ended in a boiling water bath and samples were centrifuged at 10,000 rpm for 10 min. The supernatant was then passed through a column of D152 (5 ml) at a flow rate of 0.2 ml/min and unbound compounds were discarded. The compound-bound column was washed with water (100 ml) followed by gradient elution from 0.01 M to 0.06 mM (50 ml). Every fraction was checked by HPLC-ELSD. Then, the eluates of compound **1** or **2** with higher purity were combined and lyophilized.

### GenB3-catalyzed reaction with compounds **1** and **2**

The GenB3 activity assays were conducted using mixtures containing substrate (200 μM), GenB3 (25 μM), amino donor (l-Glu, 10 mM), and PLP (1 mM) in 500 μl KPi buffer (50 mM KOH, pH 8.0). Samples were incubated at 30 °C overnight and were quenched by addition of chloroform (500 μl), followed by centrifugation to remove proteins. The supernatants were filtered through 0.22 μm microporous membranes before subjection to HPLC-ELSD analysis.

### GenP and GenB3 catalyze dideoxygenation with JI-20Ba

Enzymatic assays were conducted using 500 μl reaction mixtures containing substrate (JI-20Ba, 200 μM), GenP (15.8 μM), GenB3 (12.5 μM), ATP (10 mM), MgCl_2_ (10 mM), PLP (1 mM), α-ketoglutarate (10 mM), and Tris–HCl buffer (50 mM, pH 8.0). Samples were incubated at 30 °C overnight and were quenched by addition of chloroform (500 μl), followed by centrifugation to remove proteins. The supernatants were filtered through 0.22 μm microporous membranes before subjection to HPLC-ELSD analysis.

### HPLC-ELSD analysis of gentamicin C complexes and related intermediates

HPLC-ELSD analysis of mixtures was performed with a Welch C18 column (4.6 × 250 mm, 5 μm) connected to a SofTA Model 300 s ELSD. In the GenP-catalyzed reaction, X2, G418, JI-20A, JI-20Ba, JI-20B, and gentamicin B1 were used as substrates, and the mobile phase was 0.2 M TFA (1 ml/min). In the GenB3-catalyzed reaction, compounds **1** and **2** were used as substrates, and the mobile phase was a 92:8 mixture of 0.2 M TFA:methanol (0.8 ml/min). In the GenB4-catalyzed reaction, Ver C2a and compound **6** were used as substrates, and the mobile phase was a 92:8 mixture of 0.2 M TFA:methanol (0.8 ml/min). The mobile phase analysis of the fermentation of the strains was performed using a 98:2 mixture of 0.2 M TFA:methanol (0.8 ml/min).

### Mass spectrometry, ^1^H, and ^13^C NMR analyses

MS analysis of the molecular formulas of compounds was performed using a micrOTOF-Q operator. ^1^H and ^13^C NMR analyses of compounds were performed using a Bucker 600 MHz. Compounds were purified to 5–10 mg through the cationic resin D152 and were dissolved in 500 μl of D_2_O.

### ^31^P NMR analyses

^31^P NMR analysis of compound **2** was performed using a Bucker 400 MHz. Compound **2** was purified to 3 mg through the cationic resin D152 and was dissolved in 500 μl of D_2_O.

## Supplementary Information

**Additional file 1: Figure S1.** Sequence alignment of GenB3 with its homologs in other aminoglycoside pathways. **Figure S2.** Structure identification of compound **2**. **Figure S3.** HPLC-ELSD analysis of GenP reactions with gentamicin X2 and G418. **Figure S4.** 1H and 13C NMR of compound **6**. **Figure S5.** Ammonia analysis of GenB3-catalyzed reaction with compound **4**. **Figure S6.** GenP and GenB3 catalyze dideoxygenation. **Figure S7.** Identification of PLP-binding sites in GenB3-catalyzed reactions. **Figure S8.** Dissection of the C-4',5' reduction process catalyzed by GenB4. **Figure S9.** Schematic representation and confirmation by PCR amplification of in-frame deletions of genP and genB3 genes. **Table S1.** Kinetic constants of GenP catalyzing phosphorylation of different substrates. **Table S2.** List of primers used in this study. **Table S3.** List of strains and plasmids used in this study.

## Data Availability

The datasets and strains materials generated and analyzed during the current study are available from the corresponding author H. X. upon request.
