# Supplementary material for: Pyridoxal-5′-phosphate-dependent enzyme GenB3 Catalyzes C-3′,4′-dideoxygenation in gentamicin biosynthesis
Source: Microb Cell Fact. 2021 Mar 9;20:65. doi: 10.1186/s12934-021-01558-7 (PMC7941887; doi:10.1186/s12934-021-01558-7)
Supplement: Supplementary file 1 — Additional file 1: Figure S1. Sequence alignment of GenB3 with its homologs in other aminoglycoside pathways. Figure S2. Structure identification of compound 2. Figure S3. HPLC-ELSD analysis of GenP reactions with gentamicin X2 and G418. Figure S4. 1H and 13C NMR of compound 6. Figure S5. Ammonia analysis of GenB3-catalyzed reaction with compound 4. Figure S6. GenP and GenB3 catalyze dideoxygenation. Figure S7. Identification of PLP-binding sites in GenB3-catalyzed reactions. Figure S8. Dissection of the C-4',5' reduction process catalyzed by GenB4. Figure S9. Schematic representation and confirmation by PCR amplification of in-frame deletions of genP and genB3 genes. Table S1. Kinetic constants of GenP catalyzing phosphorylation of different substrates. Table S2. List of primers used in this study. Table S3. List of strains and plasmids used in this study. [file 12934_2021_1558_MOESM1_ESM.docx]

Supplementary information:

Pyridoxal-5'-Phosphate-dependent Enzyme GenB3 Catalyzes C-3',4'-dideoxygenation in Gentamicin Biosynthesis

**Author information**

Shaotong Zhou^a^ tong9113@126.com

Xiaotang Chen^a^ [376416460@qq.com](mailto:376416460@qq.com)

Xianpu Ni^a^* [nixianpu126@126.com](mailto:nixianpu126@126.com)

Yu Liu^a^ 1416672300@qq.com

Hui Zhang^a^ [1125235678@qq.com](mailto:1125235678@qq.com)

Min Dong^b^* mindong@tju.edu.cn

Huanzhang Xia^a^* [hzxia@syphu.edu.cn](mailto:hzxia@syphu.edu.cn)

^a^ School of Life Science and Biopharmaceutics, Shenyang Pharmaceutical University, Benxi 117004, China.

^b^ Frontiers Science Center for Synthetic Biology, Key Laboratory of Systems Bioengineering (MOE), School of Chemical Engineering and Technology, Tianjin University, Tianjin 300072, China.

These authors contributed equally: Shaotong Zhou, Xiaotang Chen

* Corresponding authors: Xianpu Ni, Min Dong, Huanzhang Xia (First corresponding author)

Table of Contents

**S1. Additional Figures**

**Figure S1.** Sequence alignment of GenB3 with its homologs in other aminoglycoside pathways.

**Figure S2.** Structure identification of compound **2**

**Figure S3.** HPLC-ELSD analysis of GenP reactions with gentamicin X2 and G418

**Figure S4.** ^1^H and ^13^C NMR of compound **6**

**Figure S5.** Ammonia analysis of GenB3-catalyzed reaction with compound **4**

**Figure S6.** GenP and GenB3 catalyze dideoxygenation

**Figure S7.** Identification of PLP-binding sites in GenB3-catalyzed reactions

**Figure S8.** Dissection of the C-4',5' reduction process catalyzed by GenB4

**Figure S9.**  Schematic representation and confirmation by PCR amplication of in-frame deletions of *gen*P and *gen*B3 genes

**S2. Additional Tables**

**Table S1.**  Kinetic constants of GenP catalyzing phosphorylation of different substrates

**Table S2.**  List of primers used in this study

**Table S3.** List of strains and plasmids used in this study

**References**

**1. Additional Figures**


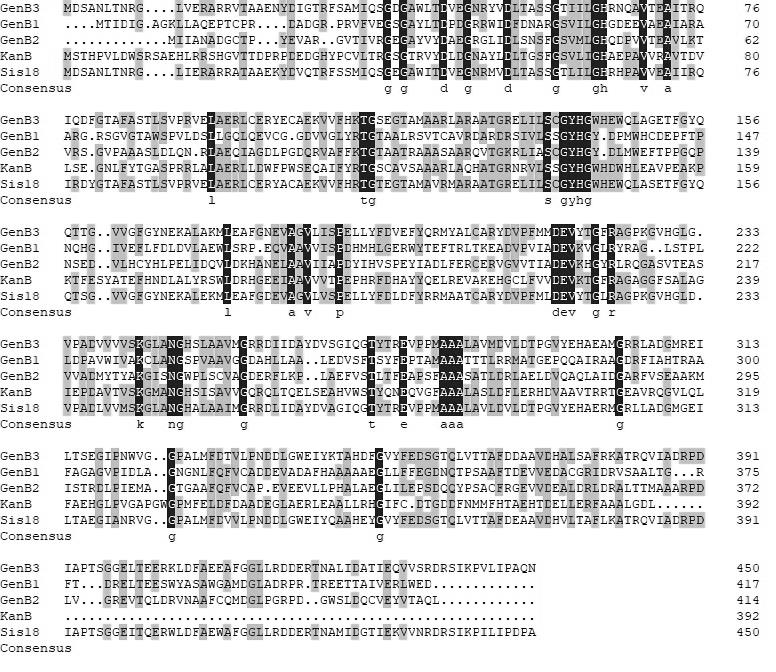


GenB3 (GenBank No. AGB13905.1), GenB1 (GenBank No. AGB13915.1), GenB2 (GenBank No. AGB13907.1): from *Micromonospora echinospora* ATCC15835, JQ975418.1; KanB (GenBank No. CAF31586.1): from *Streptomyces kanamyceticus* DSM40500, AJ628422.1; Sis18 (GenBank No. ACN38352.1): from *Micromonospora inyonensis* TS388, FJ160413.1.

**Figure S1. Sequence alignment of GenB3 with its homologs in other aminoglycoside pathways.**


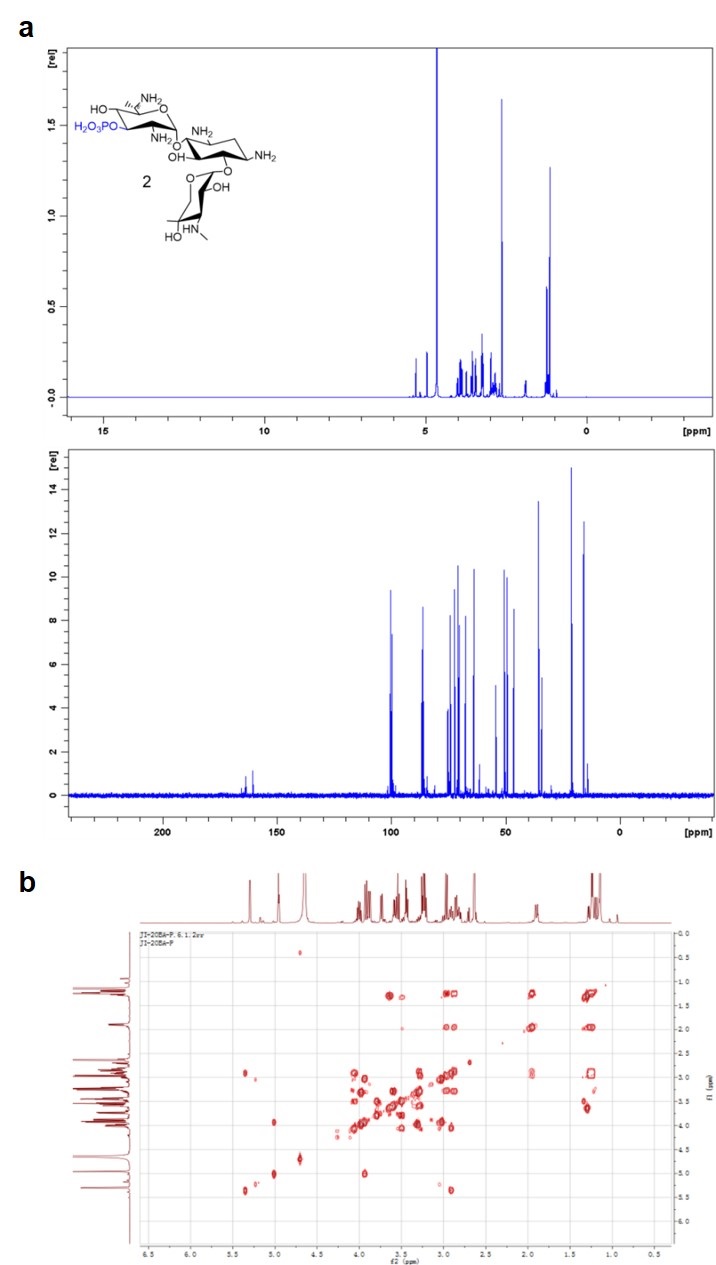


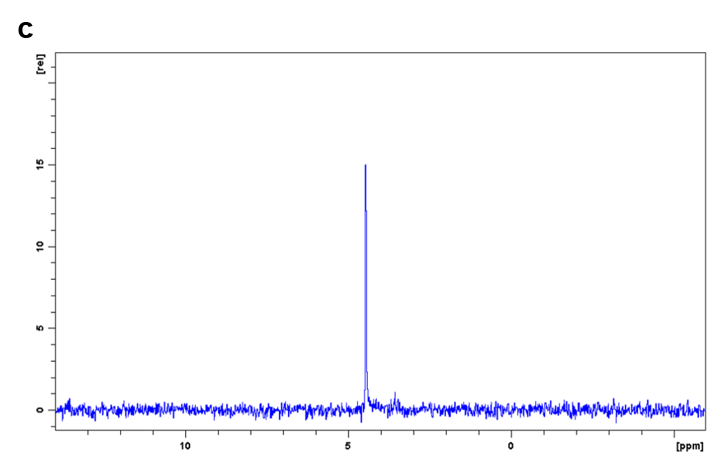


| Site | ^1^H-NMR | ^13^C-NMR |
| --- | --- | --- |
| C-1 | 3.42-3.21 (m,1H) | 50.53 |
| C-2 | 1.19(d, *J*=12.6,1H)  2.91(ddd, *J_1_*=12.3, *J_2_*=9.8, *J_3_*=4.2,1H) | 34.07 |
| C-3 | 3.42-3.21 (m,1H) | 49.19 |
| C-4 | 3.45(d, *J*=4.2,1H) | 86.5 |
| C-5 | 3.58(dd, *J_1_*=7.0, *J_2_*=2.0,1H) | 70.18 |
| C-6 | 3.54(t, *J*=9.2,1H) | 74.08 |
| C-1’ | 5.3(d, *J*=3.8,1H) | 99.64 |
| C-2’ | 2.85- 2.81 (m,1H) | 54.17(d, *J*=4.8) |
| **C-3’** | **4.00(ddd, *J_1_*=10.3, *J_2_*=8.7, *J_3_*=7.5,1H)** | **75.18 (d, *J*=5.3)** |
| C-4’ | 2.85- 2.81 (m,1H) | 72.29 |
| C-5’ | 3.74(dd, *J_1_*=10.1, *J_2_*=1.9,1H) | 85.98 |
| C-6’ | 1.9(dq, *J_1_*=13.0, *J_2_*=3.9,1H) | 46.39 |
| C-1’’ | 4.96(d, *J*=3.8,1H) | 100.18 |
| C-2’’ | 3.93(dd, *J_1_*=12.8, *J_2_*=3.4,1H) | 67.33 |
| C-3’’ | 3.42-3.21 (m,1H) | 63.83 |
| C-4’’ | - | 70.74 |
| C-5’’ | 3.88(dd, *J_1_*=10.8, *J_2_*=3.9,1H)  2.97(d, *J*=10.8,1H) | 67.42 |
| C-6’-CH_3_ | 1.24(d, *J*=6.9,3H) | 15.78 |
| C-3’’-N- CH_3_ | 2.63(s, 3H) | 35.51 |
| C-4’’- CH_3_ | 1.14(s, 3H) | 21.11 |

**Figure S2. Structure identification of compound 2.** (a) ^1^H NMR and ^13^C NMR of compound **2**. (b) HHCOSY NMR of compound **2**. (c) ^31^P NMR of compound **2** (4.49 ppm, zero point calibration is 85% phosphoric acid solution).

**
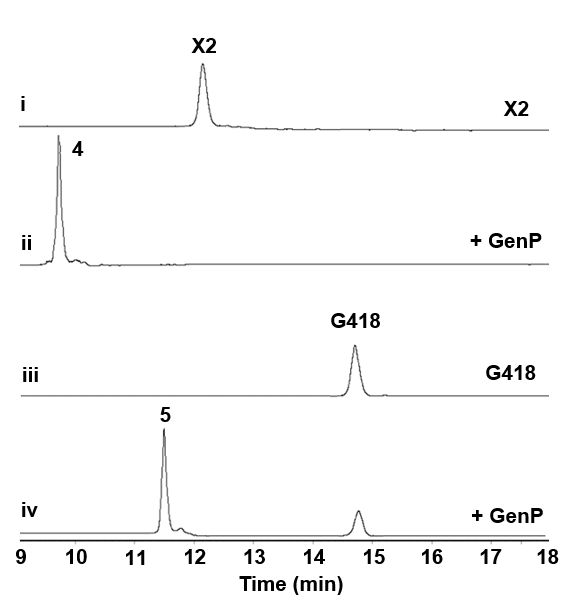
**

**Figure S3. HPLC-ELSD analysis of GenP reactions with gentamicin X2 and G418.** (i) X2 standard; (ii) GenP-catalyzed reaction with X2; (iii) G418 standard; (iv) GenP-catalyzed reaction with G418.


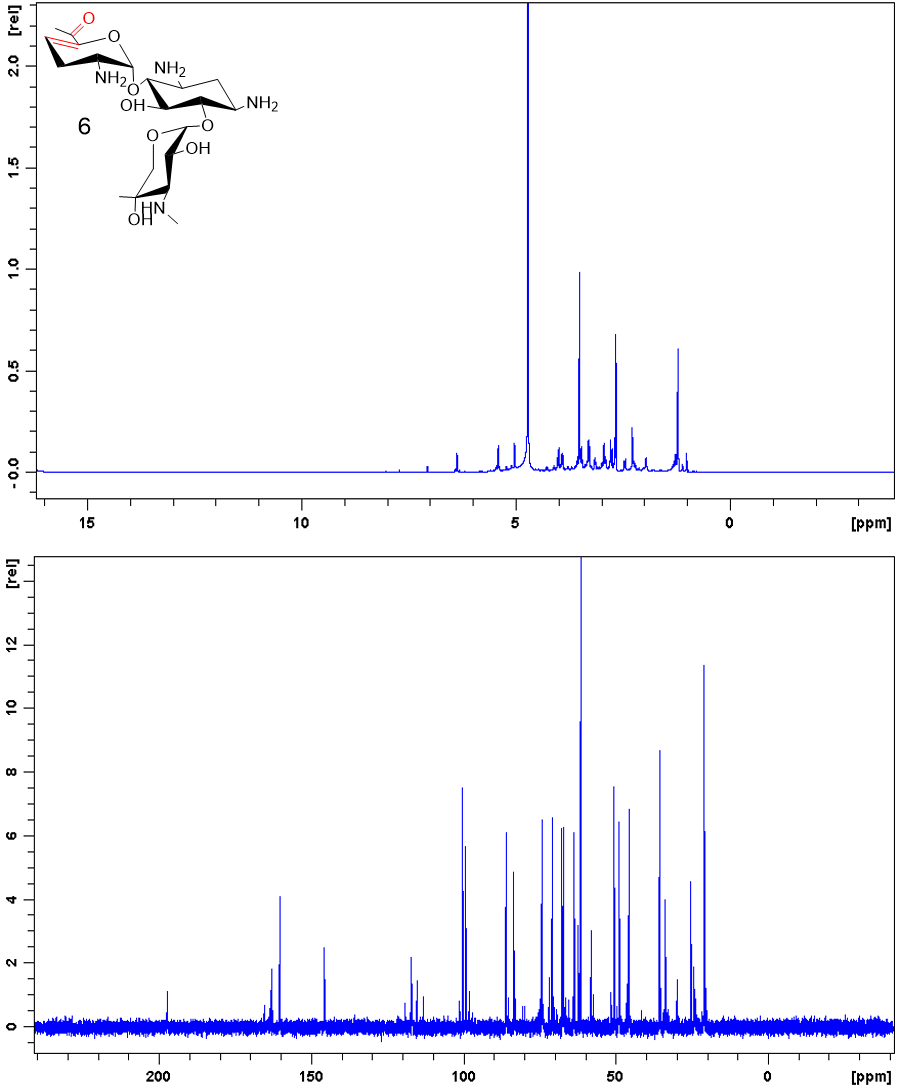


| Site | ^1^H-NMR | ^13^C-NMR |
| --- | --- | --- |
| C-1 | 3.31-3.01 (m,1H) | 50.76 |
| C-2 | 1.18 (d, *J*=12.2,1H)  2.15 (ddd, *J_1_*=10.3, *J_2_*=9.5, *J_3_*=4.0,1H) | 35.78 |
| C-3 | 3.32-3.11 (m,1H) | 49.17 |
| C-4 | 3.44 (d, *J*=4.2,1H) | 99.54 |
| C-5 | 3.55 (dd, *J_1_*=7.0, *J_2_*=2.0,1H) | 74.35 |
| C-6 | 3.23 (t, *J*=9.2,1H) | 86.31 |
| C-1’ | 5.35 (d, *J*=2.2,1H) | 100.5 |
| C-2’ | 3.10-3.02 (m,1H) | 58.16 |
| C-3’ | 1.95 (d, *J*=12.2,1H) | 29.93 |
|  | 2.20 (ddd, *J_1_*=10.5, *J_2_*=7.0, *J_3_*=4.0,1H) |  |
| C-4’ | 4.95-4.86 (m,1H) | 99.3 |
| C-5’ | - | 145.8 |
| C-6’ | - | **197.4** |
| C-1’’ | 5.06 (d, *J*=4.0,1H) | 83.7 |
| C-2’’ | 3.82 (dd, *J_1_*=10.8, *J_2_*=3.4,1H) | 72.10 |
| C-3’’ | 2.62-2.51 (m,1H) | 63.81 |
| C-4’’ | - | 71.08 |
| C-5’’ | 4.05 (dd, *J_1_*=10.8, *J_2_*=3.9,1H)  3.31 (d, *J*=10.8,1H) | 67.86 |
| C-6’-CH_3_ | 1.26 (d, *J*=6.9,3H) | 25.49 |
| C-3’’-N- CH_3_ | 2.64 (s, 3H) | 33.94 |
| C-4’’- CH_3_ | 1.16 (s, 3H) | 21.23 |

**Figure S4. ^1^H and ^13^C NMR of compound 6**

Ammonia reacts with α-ketoglutarate and NADH to produce glutamate and NAD^+^ under the action of glutamate dehydrogenase. Measuring the increase or decrease of NADH absorption at a wavelength of 340 nm can prove the production of NH_3_ in the enzyme-catalyzed reaction. Ammonia analysis reagent (10 ml solution: KH_2_PO_4_ 0.137 g, α-ketoglutarate 0.005 g, NADPH 0.002 g, ADP 0.001 g, EDTA 0.007 g, pH adjusted to 8.6 with KOH). Ammonia analysis reagent (500 μl) was mixed with 20 μl chloroform to extract the reaction solution, followed by co-cultivation at room temperature for 5 min, 2 μl glutamate dehydrogenase (70 U, 5 mg dissolved in 95 μl distilled water) was added, and UV absorption was detected at 340 nm.

It was found that the GenB3-catalyzed reaction was consistent with the change in NADH absorbance of the reaction solution without GenB3, and there was no significant decrease. Therefore, there is no generation of NH_3_ in the catalytic deamination reaction of GenB3. Combined with the change of the PLP absorption peak during the reaction process, these results prove that the amino group removed in the reaction is transferred to PLP to form PMP.


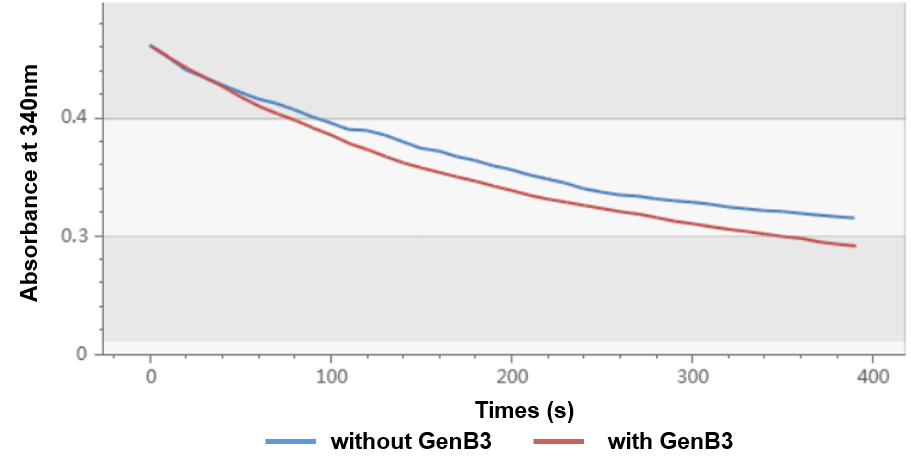


**Figure S5. Ammonia analysis of GenB3-catalyzed reaction with** **compound 4.**


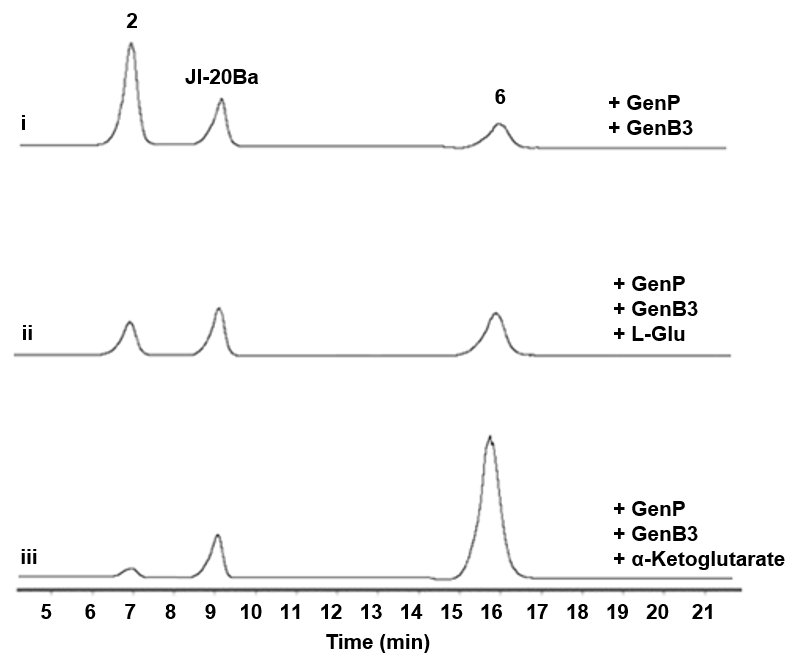


**Figure S6. GenP and GenB3 catalyze dideoxygenation.** HPLC-ELSD analysis of (i) GenP- and GenB3-catalyzed reactions, (ii) GenP- and GenB3-catalyzed reactions with L-Glu, and (iii) GenP- and GenB3-catalyzed reaction with α-ketoglutarate.


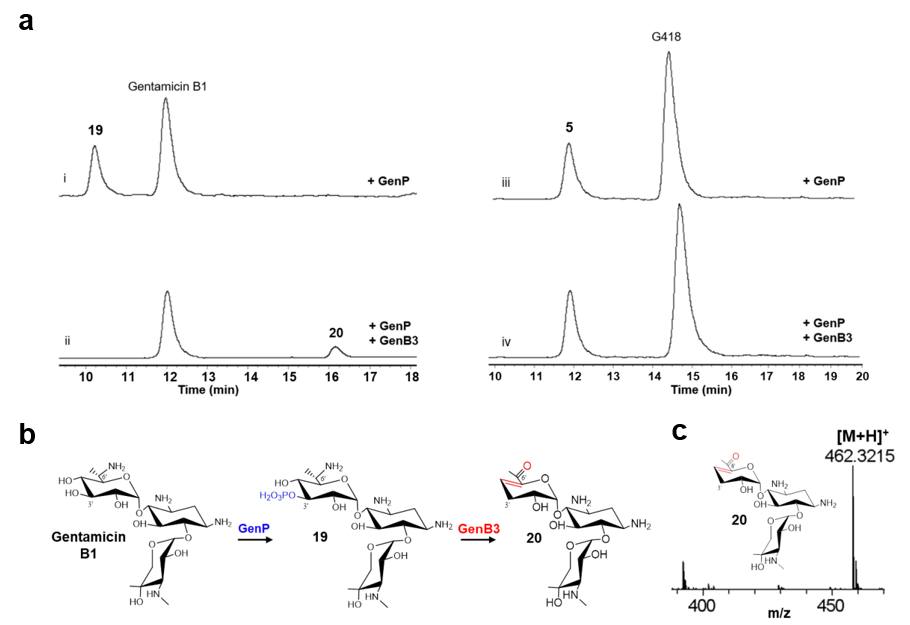


**Figure S7. Identification of PLP-binding sites in GenB3-catalyzed reactions.** a) HPLC-ELSD analysis of reactions catalyzed by the following: (i) GenP with gentamicin B1; (ii) GenP and GenB3 with gentamicin B1; (iii) GenP with G418; and (iv) GenP and GenB3 with G418. (b) Schematic of the GenP and GenB3 *in* *vitro* reactions with gentamicin B1. (c) MS analysis of compound **20**.


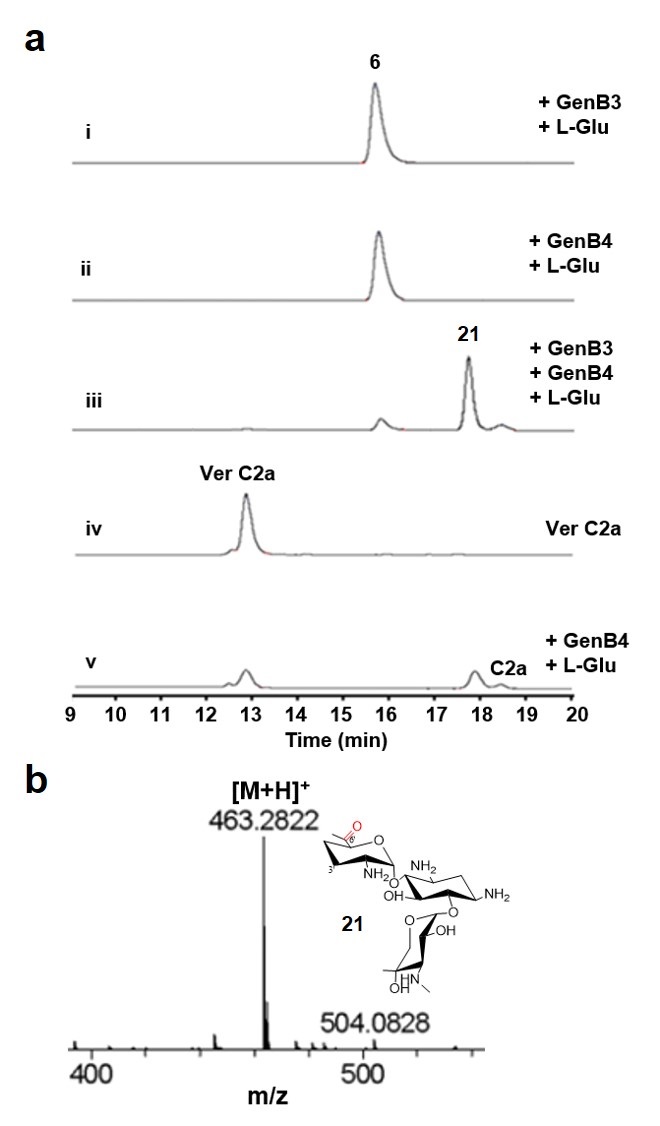


**Figure S8. Dissection of the C-4',5' reduction process catalyzed by GenB4.** (a) HPLC-ELSD analysis of GenB4-catalyzed reactions, (i) **6**; (ii) GenB4-catalyzed reaction with **6**; (iii) Ver C2a standard; (iv) GenB4-catalyzed reaction with Ver C2a. (b) MS analysis of compound **21**.

(A) Δ*gen*P

The horizontal lines indicate the size of the fragments from the wild type (2362 bp) and mutant DNA (2173 bp). PCR-amplified from DNA using primers B3P-d2 and B3P-d3.


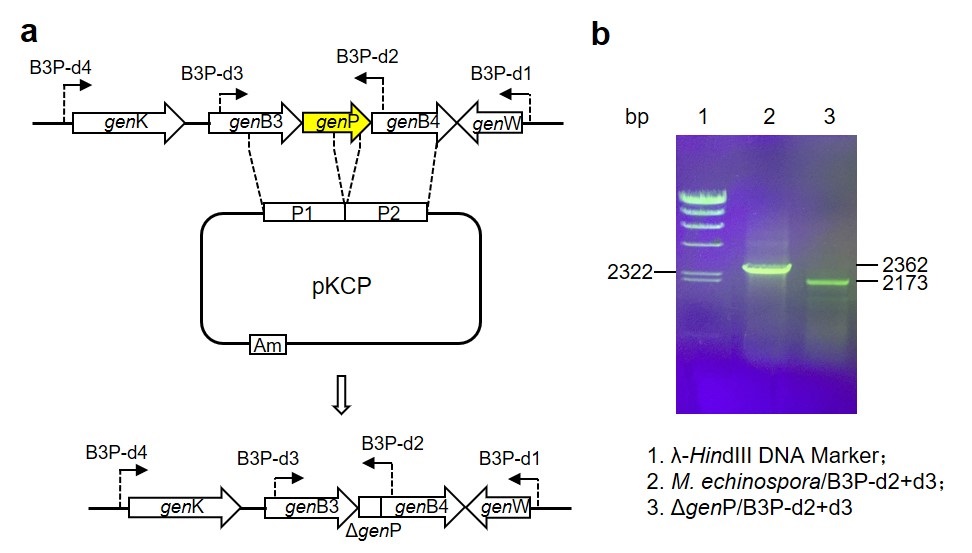


(B) Δ*gen*B3

The horizontal lines indicate the size of the fragments from the wild type (2396 bp) and mutant DNA (1187 bp). PCR-amplified from DNA using primers B3-d2 and B3-d3.

**
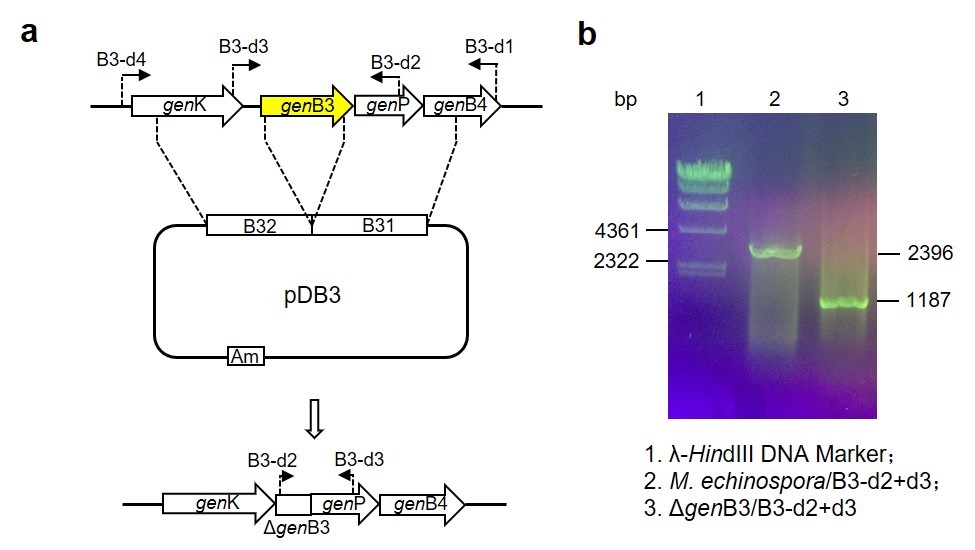
**

**Figure S9. Schematic representation and confirmation by PCR amplification of in-frame deletions of *gen*P and *gen*B3 genes.**

**S2. Additional Tables**

# Table S1. Kinetic constants of GenP catalyzing phosphorylation of different substrates

| Compound | K_m_（μmol/L） | k_cat_（/s） | k_cat_/ K_m_（L/μmol·s） |
| --- | --- | --- | --- |
| Gentamicin X2（81%） | 18.0±5.8 | 0.03±0.003 | 0.0017 |
| Gentamicin G418（89%） | 35.2±9.0 | 0.017±0.0015 | 0.0005 |
| JI-20A（98%） | 6.1±1.4 | 0.175±0.01 | 0.0287 |
| JI-20Ba（99%） | 9.4±4.3 | 0.145±0.02 | 0.0154 |
| JI-20B（86%） | 61.3±15.75 | 0.096±0.009 | 0.0016 |

# Table S2. List of primers used in this study

| Primers | Sequences (5’ to 3’) | Restriction site |
| --- | --- | --- |
| P-1 | CATATGgttgcagcaccgatacc | *Nde*I |
| P-2 | CTCGAGgctcagagaaattcgtccagcag | *Xho*I |
| B3-1 | CATATGgattctgccaacttgacg | *Nde*I |
| B3-2 | CTCGAGtcagttctgtgcggggatgag | *Xho*I |
| B4-1 | CATATGaactaccgtgagttgatcgag | *Nde*I |
| B4-2 | CTCGAGggatcagttctgtgcgggaac | *Xho*I |
| B3P-d1 | gtcgagttcgaggggaacacct |  |
| B3P-d2 | caggacatggattctgccaact |  |
| B3P-d3 | cgcgatcaccgacggatagc |  |
| B3P-d4 | ccgagatcgacgcggtgatg |  |
| B3-d1 | acgcgaacgtcgtaccgtag |  |
| B3-d2 | tcgcgctgtacagcggtca |  |
| B3-d3 | gtcgggttgaaccggtggt |  |
| B3-d4 | taccgtggagaggcacggg |  |
| B4-d1 | cgagcacctggcgacgaagatgcg |  |
| B4-d2 | ggctgacgtggtggtggtcagcaagg |  |
| B4-d3 | gccggcatcgtgcacgtcaac |  |
| B4-d4 | gcgtcaaccagcacaagctgacc |  |

# Table S3. List of strains and plasmids used in this study

| Strains or plasmids | Relevant characteristics | Reference or source |
| --- | --- | --- |
| Strains |  |  |
| *E. coli* Top 10 | Host strain for cloning | Invitrogen |
| *E. coli* ET12567/ pUZ8002 | Methylation defective, strain used in *E. coli*-*streptomyces* intergeneric conjugation | [1] |
| *E. coli* BL21(DE3) | Host strain for protein expression | Novagen |
| *M. echinospora* ATCC15836 | Wild-type strain, gentamicin C1a, C2, C2a, and C1 | This lab |
| *M. echinospora* △*gen*P | *M. echinospora* in which *gen*P was disrupted | This lab |
| *M. echinospora* △*gen*B3 | *M. echinospora* in which *gen*B3 was disrupted | This study |
| *M. echinospora* △*gen*B4 | *M. echinospora* in which *gen*B4 was disrupted | This lab |
| *M. echinospora* △*gen*B3::△*gen*B3 | Complementation of *gen*B3 in *M. echinospora* △*gen*B3 | This study |
| *M. echinospora* △*gen*B4::△*gen*B4 | Complementation of *gen*B4 in *M. echinospora* △*gen*B4 | This lab |
| *M. echinospora* △*gen*K△*gen*P | The fermentation broth is used to collect JI-20A | This lab |
| *M. echinospora* △*gen*B1△*gen*P | The fermentation broth is used to collect JI-20Ba and JI-20B | This lab |
| Plasmids |  |  |
| pIJ2925 | Cloning vector for *E. coli ori*_pUC18_, Amp^R^ | [2] |
| pD2925 | *E. coli-Streptomyces* shuttle vector, *oriT*_RP4_, *ori*_pUC18_, Amp^R^, Am^R^ | [3] |
| pKC1139 | *E. coli-Streptomyces* shuttle vector, *oriT*_SG5_, Am^R^ | [4] |
| pKCP | Gene disruption plasmid of *gen*P with pKC1139 as vector, *oriT*_SG5_, Am^R^ | This lab |
| pDB3 | Gene disruption plasmid of *gen*B3 with pD2925 as vector, *oriT*_RP4_, Amp^R^, Am^R^ | This study |
| pEAP1 | *E. coli-Streptomyces* shuttle vector, *oriT*_RP4_, *ori*_pUC18_, *int-attP*_φC31_, Amp^R^, Erm^R^ | This lab |
| pEAPP | pEAP1 containing promoter P*_hrd_*_B_, *gen*P fragment and terminator *To*, Amp^R^, Erm^R^ | This study |
| pEAPB3 | pEAP1 containing promoter P*_hrd_*_B_, *gen*B3 fragment and terminator *To*, Amp^R^, Erm^R^ | This study |
| pET28a(+) | Protein expression vector used in *E.coli*, encoding N-terminal His-tag, Km^R^ | Novagen |
| pET28aP | pET28a(+) containing *gen*P, Km^R^ | This study |
| pET28aB3 | pET28a(+) containing *gen*B3, Km^R^ | This study |

**References**

[1] Macneil DJ, Gewain KM, Ruby CL, Dezeny G, Macneil T. Analysis of Streptomyces avermitilis genes required for avermectin biosynthesis utilizing a novel integration vector. Gene 1992;111:61-8.

[2] Janssen GR, Bibb MJ. Derivatives of pUC18 that have BglII sites flanking a modified multiple cloning site and that retain the ability to identify recombinant clones by visual screening of *Escherichia coli* colonies. Gene 1993;124:133-4.

[3] Gu Y, Ni X, Ren J, Gao H, Wang D, Xia H. Biosynthesis of Epimers C2 and C2a in the Gentamicin C Complex. ChemBioChem 2015;16:1933-42.

[4] Zhihao H, Kai B, Xiufen Z, Qi Z, David AH, Tobias K, Zixin D. Repeated polyketide synthase modules involved in the biosynthesis of a heptaene macrolide by Streptomyces sp. FR-008. Mol. Microbiol. 2010;14:163-72.
